# Evaluation of nimotuzumab Fab_2_ as an optical imaging agent in EGFR positive cancers

**DOI:** 10.1038/s41598-023-37873-9

**Published:** 2023-07-07

**Authors:** Wendy Bernhard, Kris Barreto, Darien Toledo, Ayman El-Sayed, Kimberly A. Jett, Angel Casaco, Humphrey Fonge, C. Ronald Geyer

**Affiliations:** 1 Department of Pathology and Laboratory Medicine, University of Saskatchewan, College of Medicine, Saskatoon, SK Canada; 2 Center of Molecular Immunology, Havana, Cuba; 3 Department of Medical Imaging, University of Saskatchewan, College of Medicine, Saskatoon, SK Canada

**Keywords:** Molecular medicine, Diagnostic markers

## Abstract

Molecular-targeted imaging probes can be used with a variety of imaging modalities to detect diseased tissues and guide their removal. EGFR is a useful biomarker for a variety of cancers, because it is expressed at high levels relative to normal tissues. Previously, we showed the anti-EGFR antibody nimotuzumab can be used as a positron emission tomography and fluorescent imaging probe for EGFR positive cancers in mice. These imaging probes are currently in clinical trials for PET imaging and image-guided surgery, respectively. One issue with using antibody probes for imaging is their long circulation time and slow tissue penetration, which requires patients to wait a few days after injection before imaging or surgery, multiple visits and longer radiation exposure. Here, we generated a Fab_2_ fragment of nimotuzumab, by pepsin digestion and labeled it with IRDye800CW to evaluate its optical imaging properties. The Fab_2_ had faster tumor accumulation and clearance in mice relative to the nimotuzumab IgG. The fluorescent signal peaked at 2 h post injection and remained high until 6 h post injection. The properties of the Fab_2_ allow a higher signal to background to be obtained in a shorter time frame, reducing the wait time for imaging after probe infusion.

## Introduction

Molecular-targeted imaging (MTI) is becoming increasingly important for identifying, diagnosing, and treating cancers. It allows cells, which express a cancer-associated biomarker to be non-invasively detected in real time. Antibodies, which display high affinity and specificity for their targets are often used as MTI probes to detect cancer-associated biomarkers. As a therapeutic, antibodies are desirable because of their long half-lives, which allows for tri-weekly treatment regimes. For imaging however, their long half-life and slow tumor penetration requires long delays after injection before optimal images can be obtained^1^. Since antibodies take a long time to clear from the body, it reduces imaging sensitivity due to high background levels and reduces tumor specificity because of an enhanced permeability and retention effect, which can especially affect detection of low expressing biomarkers^1^.

EGFR is a useful biomarker as it is overexpressed in several cancers^2^. Anti-EGFR antibodies are used as therapeutics and more recently have been re-engineered as MTI probes, some with clinical trials currently ongoing^3^. For image-guided surgery, EGFR antibodies, like other antibodies suffer from long circulation times requiring patients to get the probe injection several days before imaging or surgery. This process can be expensive and difficult on the patient requiring extra visits and longer radiation exposure times when using probes labeled with radioisotopes. To reduce circulation times and increase tumor penetration, fragments of antibodies are often used that contain only the target binding domain and lack the antibody effector functions^4^.

Previously, we showed that the therapeutic antibody nimotuzumab can be repurposed as a PET or optical-imaging probe for EGFR positive cancers in mice^5,6^. ^89^Zr-DFO-Nimotuzumab is currently in a clinical trial to evaluate EGFR-positive lung and colon cancers using PET (*NCT04235114)*. The IRDye800CW-nimotuzumab probe is currently in a clinical trial for image-guided surgery for lung cancer (*NCT04459065)*. Both nimotuzumab imaging probes have a peak signal at or after 72 h post injection in mice. Based on other anti-EGFR imaging probes, there is typically a 48–96 h wait after infusion in humans prior to imaging. In this study, we characterized the imaging properties of a Fab_2_ fragment of nimotuzumab labelled with the fluorescent dye IRDye800CW as a potential probe for image guided surgery.

## Results

### Synthesis of nimotuzumab Fab_2_

**Figure 1:**
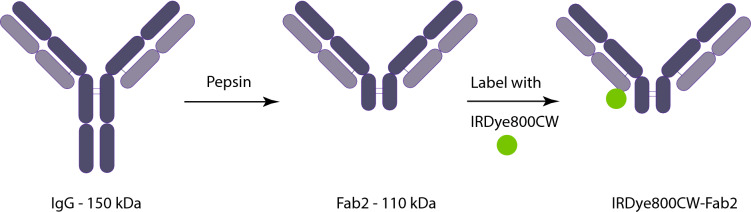
Nimotuzumab IgG pepsin digestion and Fab_2_ labeling. Nimotuzumab IgG (150 kDa) was digested with pepsin resin. The Fab_2_ (110 kDa) was purified with Pierce F(ab′)_2_ Preparation Kit followed by size exclusion and labeled with IRDye800CW.

**Figure 2:**
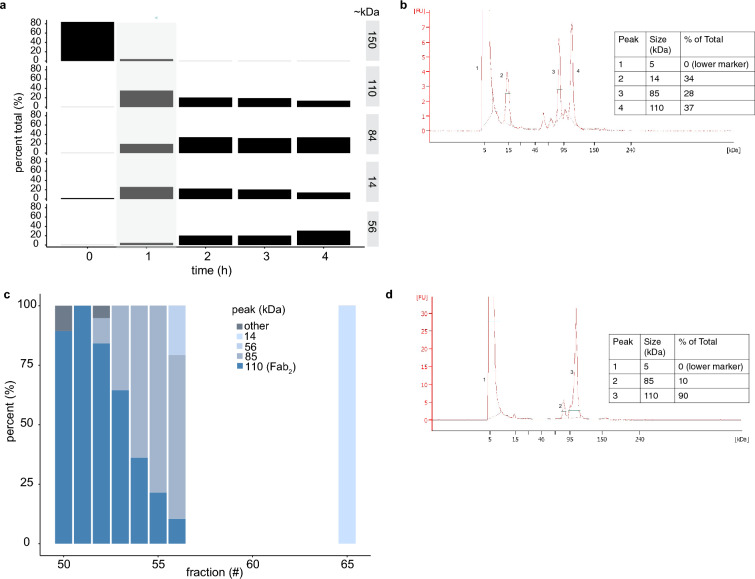
Analysis of Nimotuzumab pepsin digestion. (**a**) The percentage of IgG or fragments at different pepsin digestion time points are shown on the left y-axis and the sizes (kDa) are shown on the right y-axis. The column highlighted in grey shows the optimal time for pepsin digestion, which yields the most Fab_2_. The pepsin digestion time in hours (h) is shown on the x-axis. (**b**) Electronic electrophoresis analysis of Fab_2_ after pepsin digest and protein A clean up for IgG and Fc fragments. The peaks are labeled numerically, the first peak shows the 5 kDa lower marker, protein sizes are shown in kDa and the percent (%) of the total protein is shown. (**c**) Size exclusion analysis and purification of Fab_2_ after pepsin digest of each fraction. The different colors represent the percentage of each fragments size from each fraction. (**d**) Electronic electrophoresis analysis of the final Fab_2_ preparation. After pepsin digest, protein A clean up of IgG and Fc fragments, and size exclusion purification (collection of fractions 50–52).

To produce the nimotuzumab Fab_2_, we digested the nimotuzumab IgG with pepsin (Fig. 1). During digestion, samples were taken every hour for 4 h to determine the optimal digestion time (Fig. 2). Several reaction products were observed including: a 150 kDa product corresponding to the size of the undigested IgG, a 109 kDa fragments, which corresponded to the molecular weight of the Fab_2_, ~ 80, a 14 kDa fragment, and a 56 kDa fragment which corresponds to the size of a Fab fragment. The highest yield of Fab_2_ was obtained after 1 h of digestion. After this time point, there was a decrease in the amount of Fab_2_ and an increase in smaller fragments (Fig. 2a). After pepsin digestion for 1 h, the Fab_2_ fragment was purified by running through a protein A column to bind the contaminating IgG and Fc fragments. The Fab_2_ was dialyzed with PBS, yielding a purity of 37% (Fig. 2b). The Fab_2_ was further purified by size exclusion (Fig. S1). Fractions 50–56 and 65 were collected and analyzed for size and purity (Fig. 2c). Fractions 50–52 were considered significantly pure and were pooled, resulting in a Fab_2_ with a purity of 90% (Fig. 2d).

### Labeling, binding properties, and stability of nimotuzumab Fab_2_

**Figure 3:**
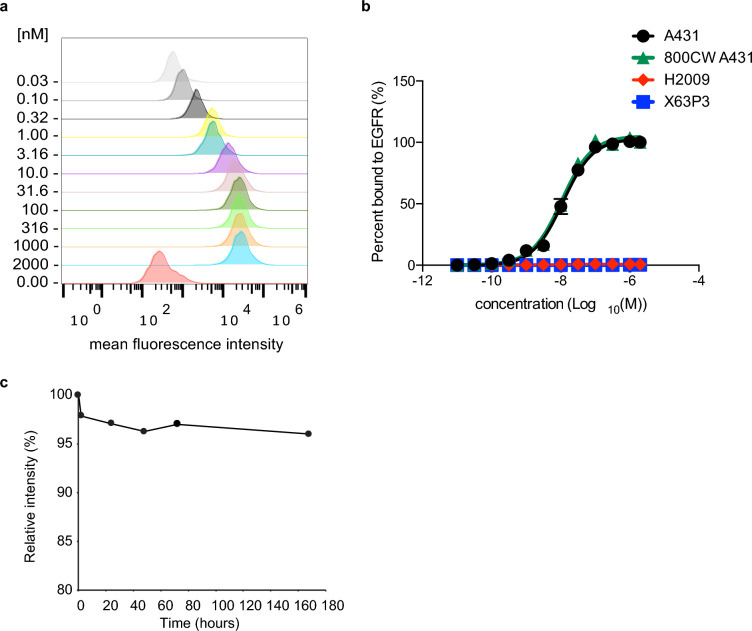
Binding of nimotuzumab Fab_2_ to cells and serum stability. Fab_2_ and IRDye800CW-labeled Fab_2_ were titrated with A-431 cells and analyzed by flow cytometry to obtain a binding constant to EGFR. (**a**) shows a representative histogram of the mean fluorescence intensity (MFI) of the unlabeled Fab_2_ at each concentration tested. (**b**) shows the titration curves produced from plotting the concentration vs normalized MFI to obtain percent bound. (**c**) serum stability of IRDye800CW-labeled Fab_2_ was tested at 37 °C, run on SDS-PAGE and analyzed for IRDye800CW fluorescence.

The nimotuzumab Fab_2_ was labeled non-specifically on primary amines with IRDye 800CW NHS ester (Fig. 1) to give a labeling ratio of 1–2 IRDye800CW molecules per Fab_2_. Binding of the IRDye800CW-Fab_2_ and the Fab_2_ were characterized using flow cytometry with A-431 cells, an EGFR positive human epidermoid carcinoma cell line. IRDye800CW labeling did not affect the binding of the Fab_2_ as the Fab_2_ had a K_D_ of 11 ± 1 nM and the IRDye800CW-Fab_2_ had a K_D_ of 11 ± 2 nM (Fig. 3). Also, the K_D_ of the Fab_2_ was not significantly different (p > 0.05) than the K_D_ of nimotuzumab IgG of 9.7 ± 1.6 nM^6^. The Fab_2_ did not bind to either P3X63Ag8, a mouse plasmacytoma cell line or H2009, a human stage 4 adenocarcinoma cell line.

The stability of the Fab_2_ was tested in human serum at 37 °C and was stable for one week (Fig. 3c). Samples of the Fab_2_ were collected and analyzed by SDS-PAGE and then scanned on the LI-COR Odyssey to determine the amount of IRDye800CW labeled Fab_2_ at 800 nm. After 2 h the mean fluorescence intensity (MFI) of Fab_2_ dropped to 97% and stayed at 97% for three days. After a week in serum at 37 °C the Fab_2_ was stable with an MFI of 96% compared to the starting amount.

### Optical imaging of nimotuzumab Fab_2_

**Figure 4:**
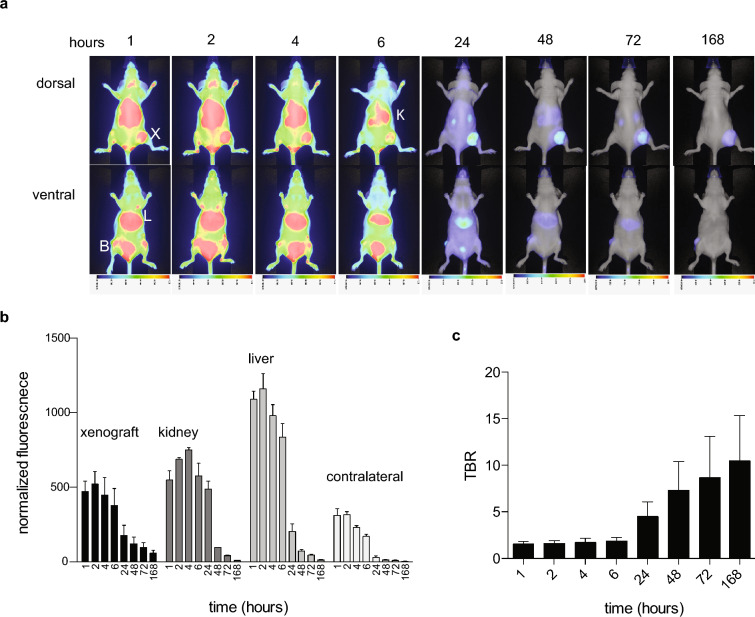
Optical imaging of IRDye800CW-labled nimotuzumab Fab_2_ in A-431 xenografts. Mice bearing A-431 xenografts were injected with IRDye800CW-labeled nimotuzumab Fab_2_ and imaged over time. (**a**) Dorsal and ventral mouse images taken over time. X = xenograft, B = bladder, L = liver, K = kidney. (**b**) Normalized signal of xenograft, kidney, liver and contralateral over time. (**c**) Tumor-to-background ratio (TBR) in the xenograft over time.

**Figure 5:**
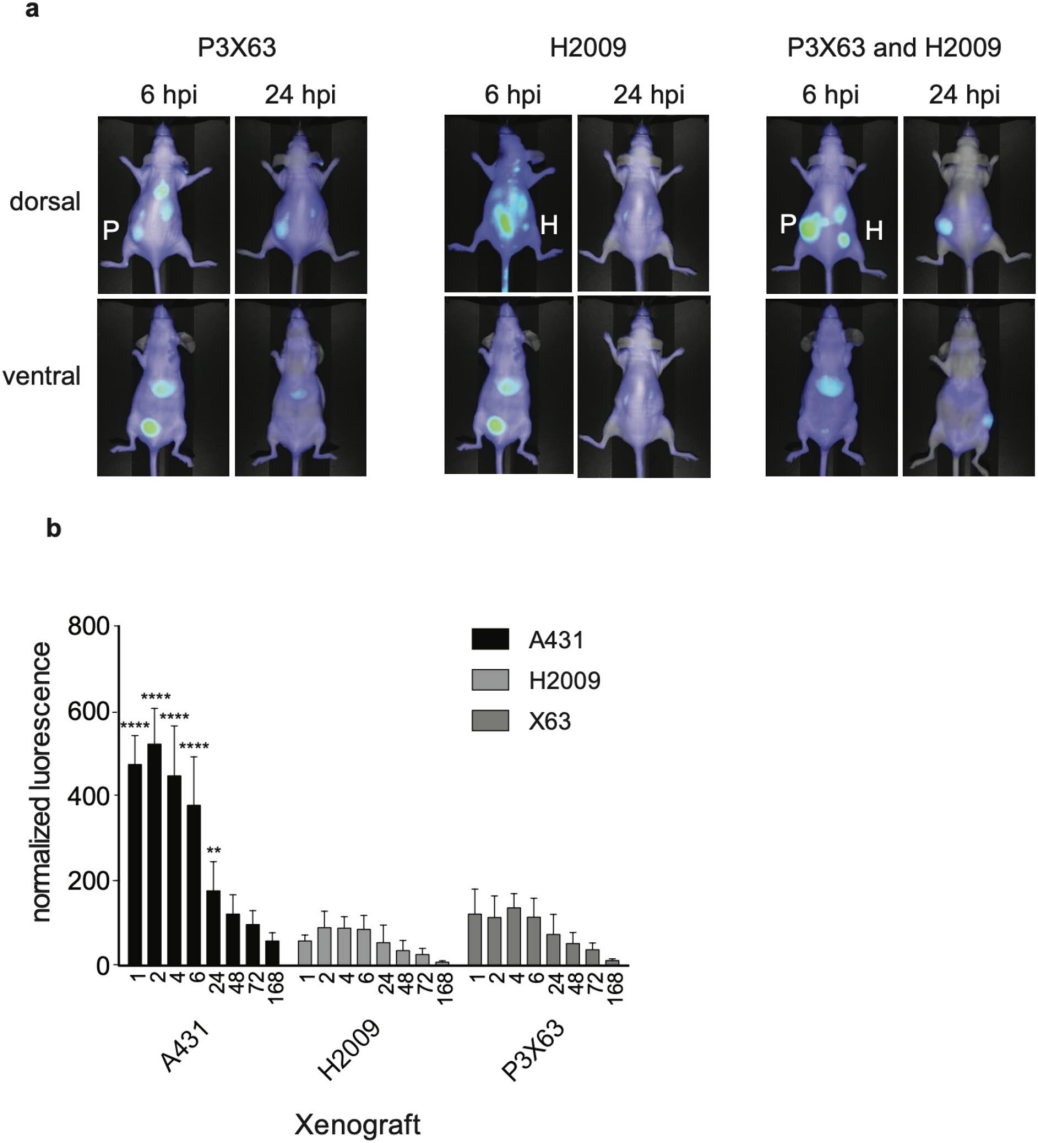
Optical imaging of IRDye800CW-labled nimotuzumab Fab_2_ in control xenografts. Mice bearing H2009 and/or P3X63Ag8 xenografts were injected with IRDye800CW-labeled nimotuzumab Fab_2_ and imaged over time. (**a**) Dorsal and ventral mouse images taken, 6 and 24 h images are shown. P = P2X63, H = H2009. (**b**) Normalized signal of control xenografts compared to A-431 EGFR positive xenografts are shown over time. ****p ≥ 0.0001, **p ≥ 0.01.

Nimotuzumab Fab_2_ labeled with IRDye800CW was injected into CD-1 nude mice bearing A-431, P3X63Ag8, and/or H2009 xenografts and imaged over time. The Fab_2_ accumulated in the EGFR positive A-431xenograft (MFI = 483 ± 98) after 1 h and peaked 2 h after injection with an MFI of 515 ± 100 (Fig. 4a,b). After 2 h, the Fab_2_ started to clear from the xenograft. Despite the quick clearance of the Fab_2_ it was still visible in the xenograft after 168 h. The Fab_2_ cleared quickly through the liver and kidney (Fig. 4a,b). It was also visible in the bladder at the early timepoint (Fig. 4). The tumor to background ratio (TBR) for the Fab_2_ was approximately 2 after 2 h (Fig. 4c). The Fab_2_ TBR remained around 2 until 24 h where it increased to over 4. After 48 h the TBR was over 10 and continued to increase until 168 h post injection. There was minimal accumulation in both EGFR negative control xenografts where the highest MFI for P3X63Ag8 was 136 ± 33 and H2009 was 90 ± 39 (Fig. 5).

### Comparison of in vivo imaging of IRDye800CW-labeled nimotuzumab IgG and Fab_2_

**Figure 6:**
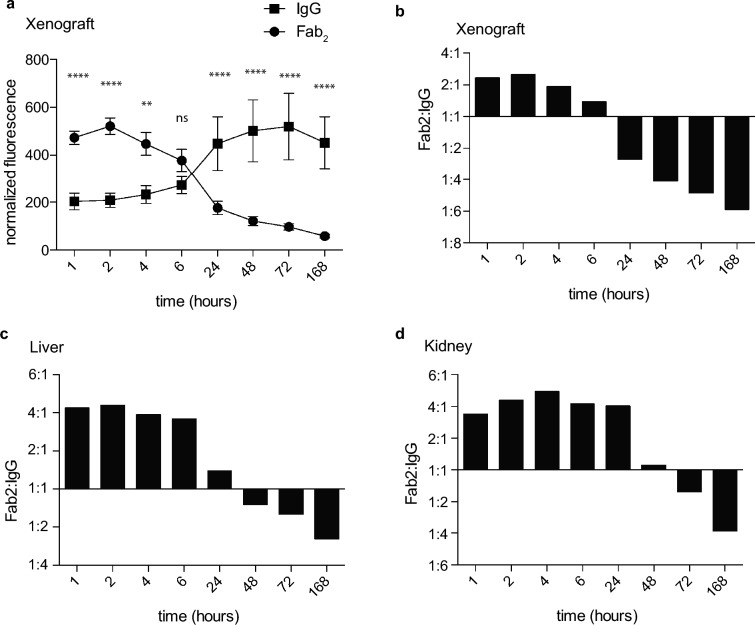
Comparison of nimotuzumab IgG and Fab_2_ imaging in mice. (**a**) Normalized signal of IRDye800CW-labeled nimotuzumab IgG and Fab_2_ imaging in the xenograft. The ratio of fluorescent signal of nimotuzumab Fab_2_ to IgG over time in the xenograft (**b**), liver (**c**), and kidney (**d**) Errors are shown as standard deviation. Data from nimotuzumab IgG was previously published in^6^ and was re-analyzed and presented here as a comparison to the Fab_2_. Significance shown on A-431 xenografts is compared to both controls. ****p ≥ 0.0001, **p ≥ 0.01.

We compared the imaging properties of the nimotuzumab Fab_2_ and IgG^6^. At 6 h post injection the Fab_2_ and IgG had similar fluorescent signals in the xenograft (Fig. 6a). The fluorescent signal of the Fab_2_ in the xenograft is significantly higher than the IgG at the early time points with twice the amount of signal at 1, 2, and 4 h post injection, while the IgG was higher at time points after 6 h (Fig. 6a,b). The Fab_2_ MFI was 473 ± 68, 521 ± 84, 447 ± 117 at 1, 2, and 4 h post injection, respectively, which was statistically higher than the MFI of the IgG at the same time points, which were 204 ± 35, 208 ± 30, and 232 ± 37 (Fig. 5a). The Fab_2_ cleared through the liver and kidney within the first 6 h and had 4–5 times the signal in liver and kidney compared to the IgG (Fig. 6c,d).

## Discussion

Nimotuzumab is an anti-EGFR IgG that is used as a therapeutic for EGFR positive cancers in a number of jurisdictions^7^. EGFR optical probes are useful for guiding tumor resection^8^. We recently constructed an IRDye800CW-labeled nimotuzumab for fluorescent image guided surgery of EGFR positive xenografts in mouse models^6^ and have since moved this probe to clinical trials. Fluorescence of the IRDye800CW-nimotuzumab probe peaks in imaging at 96 h post injection in mice, however the optimal time point for imaging in humans is currently unknown. By comparing other IRDye800CW-EGFR binding antibodies, the optimal time is estimated to be between 4 and 6 days post injection^1^. We characterized the imaging properties of the Fab_2_ nimotuzumab to see if this probe had faster time to maximum fluorescence while still maintaining a high tumor-to-background ratio (TBR).

We constructed the Fab_2_ by digesting nimotuzumab with pepsin. Following digestion, the Fab_2_ required two purification steps to obtain 90% purity. A-431 cell line was used in this study as a positive cell line since it has high EGFR expression. The Fab_2_ accumulated in the EGFR positive xenograft (A-431), significantly more than the control xenografts (P3X63Ag8 and H2009). P3X63Ag8 is a murine cell line that expresses EGF, however, nimotuzumab does not bind to murine EGFR. The H2009 cell-line is human cell line with low levels of EGFR. We showed these controls together and alone in a mouse xenograft model have low accumulation of the Fab_2_. Both control xenografts had significantly lower accumulation of the Fab_2_ compared to the EGFR positive xenograft A-431.

Every probe has its own set of limitations. The appropriate probe should be evaluated based on the application. The purpose of the Fab_2_ optical probe was to demonstrate specific targeting to xenografts expressing the EGFR receptor. A variety of cancers overexpress EGFR^2^. This probe could therefore be used to determine EGFR expression levels in vivo, overcoming limitations of punch biopsies, which due to their small size do not represent the whole tumor. Whether a tumor expresses EGFR or not could be used to stratify patients for anti-EGFR antibody therapies such as cetuximab, panitumumab, and nectitumumab**.** This information is not provided by FDG, which is taken up by metabolically active cells and detects non-cancerous cells, including lymphocytes^9^. In addition, metabolically inactive tumors are not detected by FDG. Based on previous studies, we have seen molecular weight cut-offs for renal versus hepatic clearance. Probes with molecular weights in between the extremes of the renal and hepatic cutoffs are observed to undergo clearance through both kidney and liver. This is a limitation of the probe. However, we did not evaluate the signal-to-noise of a metastatic tumor in the liver or kidney to determine if the probe would be visual tumors in this region. Regardless of this, smaller imaging probes tend to clear quicker than larger proteins such as IgGs giving them less time to bind to their target^1^. In future experiments to reduce the signal in the liver and kidney, blocking experiments could be attempted by injecting a cold dose of the probe. Also, a dose escalating study could be done to optimize xenograft uptake.

Compared to the imaging properties of nimotuzumab IgG^6^, the Fab_2_ fluorescent signal was higher than the IgG at the earliest time points (1–4 h post infection), however after 24 h post injection the Fab_2_ cleared quickly as the IgG signal continued to increase. The nimotuzumab IgG binding was previously extensively characterized in a number of cell lines^6^. The Fab_2_ was visible in the tumor as early as 1 h after injection and was still visible at 168 h, which is not the case with control antibody fragments of this size^6^. The overall signal in the tumor of the Fab_2_ was higher than that of the control IgG previously tested in A-431 xenografts at early time points^6^. A control IgG was also tested in the A-431 cell line and did not accumulate implying that any accumulation would be due to specific binding of the Fab_2_ and not from random distribution such as the EPR effect. The nimotuzumab Fab_2_ cleared quickly through the liver, kidney and bladder in the first few hours after injection. Overall, the Fab_2_ had desirable properties for an optical imaging probe since it peaked early with maximum fluorescence as early as 2 h post injection. This rapid accumulation in the tumor is desirable for imaging as Fab_2_ could be injected and imaged the same day.

These results are similar to other results seen with Fab_2_ imaging probes. The Fc domain present on IgGs contributes to their long half-life due to their interaction with the neonatal Fc receptor, which results in IgG recycling^10^. Previous studies have shown that IgGs clear slower than Fab_2_, which clear slower than Fab^11,12^. Smaller antibody fragments clear faster, which reduces background signal, but it also results in decreased tumor uptake^10^. The faster clearance also results in lower exposure of the imaging probe to normal tissues, which will potentially decrease toxicity^13^. Relative to the Fab_2_, Fabs clear even faster, however they are limited by their mono-valency, which decreases their affinity, relative to a Fab_2_ or IgG^14,15^.

Previous Fab_2_ studies have been reported with the anti-EGFR antibody panitumumab Fab_2_ labeled with ^111^In, ^86^Y^13^, or ^64^Cu^16^. The panitumumab Fab_2_ worked well as an imaging agent, however no time points before 24 h were provided for comparison^13,15^. Cetuximab, another anti-EGFR antibody was digested to a Fab_2_ and labeled with ^111^In in two different studies and tested for theranostic properties. Imaging with ^111^In-labeled Fab_2_ showed accumulation in the tumor that peaked at 24 h post injection^17,18^. Two other studies using ^64^Cu-cetuximab Fab_2_ only reported images at 24 h and showed the ability of the Fab_2_ imaging probe to monitor treatment^19,20^. The quick clearance of the Fab_2_ will result in less radiation exposure if used as a PET or SPECT imaging probe allowing it to be injected for monitoring purposes multiple times over the course of treatment if necessary. In addition to quicker xenograft accumulation compared to the other EGFR Fab_2_, nimotuzumab IgG also has less toxicity when used as a therapeutic compared to other EGFR antibodies^21^ which makes its Fab_2_ an attractive imaging candidate. In summary, this study supports nimotuzumab Fab_2_ as a promising imaging probe for EGFR-positive tumors.

## Methods

### Nimotuzumab pepsin digestion of IgG, Fab_2_ purification, and analysis

Twenty mg of nimotuzumab (Center of Molecular Immunology, Cuba) was digested and purified with Pierce F(ab′)_2_ Preparation kit (ThermoFisher Scientific), according to the manufacturer’s instructions. Samples were collected every hour to analyze for digestion. The purified Fab_2_ was dialyzed in PBS overnight and was further purified using a HiLoad 16/600 Superdex 200 pg size exclusion column (GE Health Care Life Sciences) with the AKTAprime plus FPLC system. Fractions 50–52 (Fig. 2) were pooled and five µg of protein was analyzed on an Agilent Bioanalyzer 2100.

### Protein labeling and stability

One mg of Fab_2_ was labeled with IRDye800CW NHS ester (LI-COR Biosciences) for optical imaging experiments as previously report^6^. The Fab_2_ was labeled at a 1:3 protein:dye ratio in PBS at 4 °C overnight on a rotator. Free dye was removed by size exclusion with a Zeba Spin Desalting Column 7 MWCO. The final Fab_2_ buffer was PBS. Concentration and labeling ratio were determined as previously reported^6^.

Stability of the nimotuzumab Fab_2_ labeled with IRDye800CW as tested by incubating IRDye800CW-Fab_2_ in human serum. This study was conducted according to the guidelines of the Declaration of Helsinki and approved by the Institutional Review Board of the University of Saskatchewan (protocol # Bio 16-275) for collection of blood from healthy donors. Informed consent was obtained from the donor. Blood was collected and allowed to clot at room temperature for 15 min. Serum was collected by centrifuging the clotted blood at 1500×*g* for 10 min. IRDye800CW labeled Fab2 was added to human serum at a concentration of 0.2 mg/mL and incubated at 37 °C. Samples were collected at 0, 2, 24, 48, 72 and 168 h, run on sodium dodecyl sulphate polyacrylamide gel electrophoresis (SDS-PAGE) and imaged on the LI-COR Odyssey CLx scanner on the 800 nm channel. The relative fluorescence intensity of each time point was normalized to the 0 h band intensity.

### Tissue culture and flow cytometry

The EGFR positive epithelial cancer cell line A-431 and EGFR negative cell lines P3X63Ag8, a mouse plasmacytoma cell line and H2009, a human stage 4 adenocarcinoma were purchased from ATCC. A-431 and P3X63Ag8 were cultured in 90% Roswell Park Memorial Institute medium (RPMI) with 10% fetal bovine serum (FBS) in a humidified incubator with 5% CO_2_. H2009 cells were cultured in DMEM:F12 medium with 0.005 mg/ml insulin, 0.01 mg/ml transferrin, 30 nM sodium selenite, 10 nM hydrocortisone, 10 nM beta-estradiol, 4.5 mM l-glutamine and 5% FBS.

For flow cytometry cells were treated with trypsin, washed, and suspended in PBSF (phosphate buffered saline and 2% fetal bovine serum). The Fab_2_ was titrated (0–2 µM) with 1 × 10^5^ cells, incubated for 30 min at room temperature and for 15 min on ice. Cells were washed and incubated with a 1:50 dilution of FITC labeled Goat F(ab′)2 fragment anti-human IgG (H + L) antibody (Beckman Coulter) for 30 min on ice. Cells were washed, suspended in PBSF, and analyzed on a Beckman Coulter Gallios flow cytometer. Mean fluorescent intensity (MFI) values were collected using FlowJo version 10.5.3. MFI values were normalized then analyzed using a non-linear regression curve fit to obtain K_D_ values using GraphPad Prism version 6.

### Mouse xenograft models and optical imaging

All mouse experiments were performed with approval and under the supervision and guidelines of the University of Saskatchewan Animal Care Committee and ARRIVE. All applicable institutional and/or national guidelines for the care and use of animals were followed. Female CD-1 nude mice were obtained from Charles River Canada. All mice had ad libitum access to food and water. Mice 6–8 weeks old were used for experiments. Mice were anesthetized with 3% isoflurane for xenograft implantation. Mice were randomly divided into two groups (4 mice/group) to receive A-431 or P3X63Ag8 and H2009. To conserve mice the negative control cells were grafted into the same mouse. Ten million A-431 or P3X63Ag8 and H2009 cells were washed and suspended in a 1:1 mixture of serum free RPMI and Corning Matrigel Matrix (Corning) on ice. The cell-matrigel mixture of the different cell lines was injected subcutaneously into the right (A-431 or H2009) or left (P3X63Ag8) hind flank of CD-1 nude female mice. Xenografts were monitored with external calipers until they reached a size of 150–300 mm^3^. Animals were included in the study if a xenograft of this size was established. At this point, the mice were injected with 0.5 nmoles of IRDye800CW-Fab_2_. Mice were anesthetized with 3% isoflurane and imaged using a LI-COR Pearl small animal imaging system. Mice were imaged at 1, 2, 4, 6, 24, 48, 72 and 168 h. Confounders were not controlled and group allocation was not blinded.

For analysis of imaging probe accumulation, at least three mice were analyzed per xenograft type for A-431, the positive control and for the negative controls, P3X63Ag8 and H2009. Three regions of interest (ROI) were selected from equal sized areas containing the same number of pixels for xenografts, liver, kidney, and contralateral side. The MFI of ROIs were averaged, then normalized by labeling ratio. The data was tested for normal distribution using the D’Agostino & Pearson ominibus K2 normality. Both the Fab_2_ and IgG pass the normality test with p > 0.05. A two-way analysis of variance (ANOVA) with multiple comparisons using GraphPad Prism version 6 was used to compare the K_D_ values, normalized fluorescent signals, and tumor-to-background ratios (TBR) for accumulation of the Fab_2_. All experiments were performed with at least three biological replicates (n ≥ 3), A-431 group had 3 mice, P3X63Ag8 had 4 mice and H2009 had 3 mice. The error represents standard error of the mean (SEM).

## Supplementary Information

Supplementary Legends.

Supplementary Figure S1.

## Data Availability

All data generated or analyzed during this study are included in this published article.
