# Supplementary material for: Evaluation of nimotuzumab Fab2 as an optical imaging agent in EGFR positive cancers
Source: Sci Rep. 2023 Jul 7;13:10990. doi: 10.1038/s41598-023-37873-9 (PMC10328982; doi:10.1038/s41598-023-37873-9)
Supplement: Supplementary file 1 — Supplementary Legends. [file 41598_2023_37873_MOESM1_ESM.pdf]

Figure S1. Size exclusion chromatography purification of nimotuzumab Fab2.

Nimotuzumab Fab2 was further purified by size exclusion after purification on a protein A column. The peak on the left is the Fab2 and the peak on the right is the IgG. Fractions were collected and analyzed by bioanalyzer and fractions 50-52 were used in flow cytometry and imaging experiments.
