# Supplementary figures and images for: Evaluation of nimotuzumab Fab2 as an optical imaging agent in EGFR positive cancers
Source: Sci Rep. 2023 Jul 7;13:10990. doi: 10.1038/s41598-023-37873-9 (PMC10328982; doi:10.1038/s41598-023-37873-9)

Fig. S1

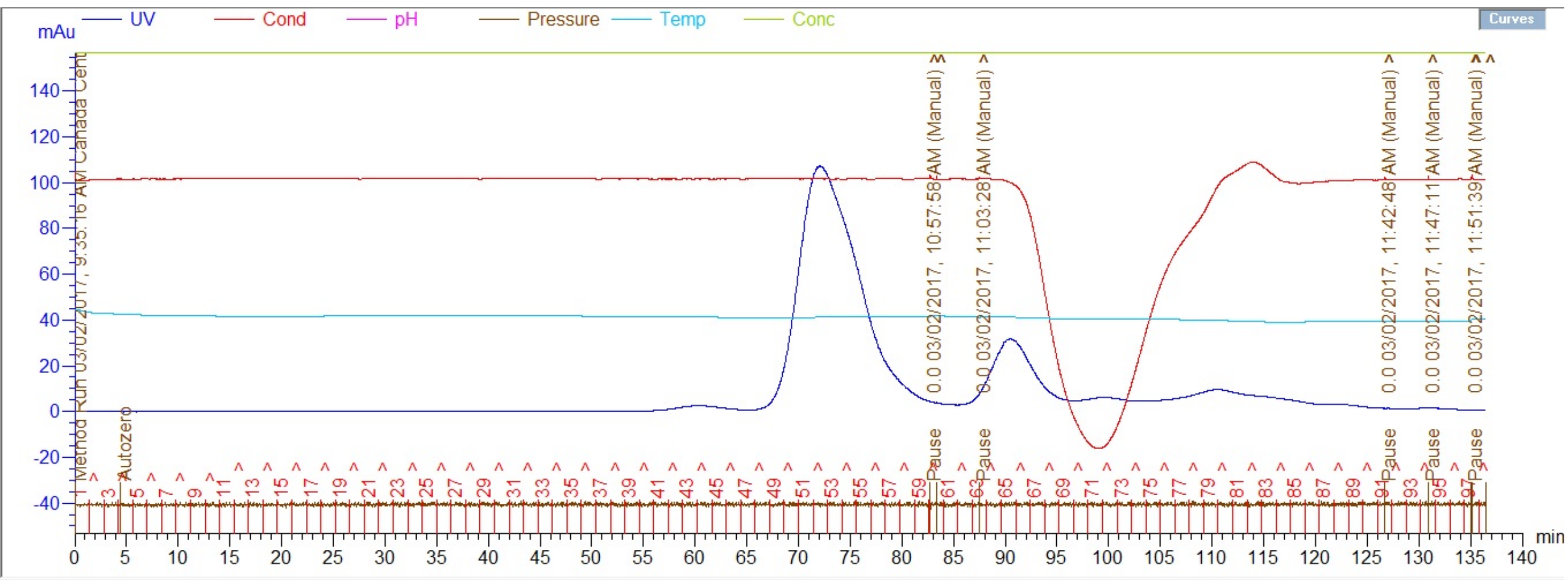

Supplement: Supplementary file 2 — Supplementary Figure S1. [file 41598_2023_37873_MOESM2_ESM.pdf]
